# Immunohistochemical evaluation of bone regeneration induced by human umbilical cord mesenchymal stem cells around implants in an osteoporotic rat model

**DOI:** 10.1016/j.jds.2025.08.042

**Published:** 2025-09-09

**Authors:** Mefina Kuntjoro, Nike Hendrijantini, Michael Josef Kridanto Kamadjaja, Bambang Agustono Satmoko Tumali, Jennifer Widjaja, Eric Priyo Prasetyo, Guang Hong

**Affiliations:** a Department of Prosthodontics, Faculty of Dental Medicine, Universitas Airlangga, Surabaya, Indonesia; b School of Dental Medicine, Universitas Ciputra, Surabaya, Indonesia; c Department of Conservative Dentistry, Faculty of Dental Medicine, Universitas Airlangga, Surabaya, Indonesia; d Division for Globalization Initiative, Liaison Center for Innovative Dentistry, Graduate School of Dentistry, Tohoku University, Sendai, Japan

**Keywords:** Medicine, Mesenchymal stem cells, Osseointegration, Osteoporosis, Postmenopause, Umbilical cord

## Abstract

**Background/purpose:**

Osteoporosis in the jawbone can compromise the success of dental implant treatment. Human umbilical cord mesenchymal stem cells (hUCMSCs) have demonstrated osteogenic differentiation potential. This study aimed to evaluate the effect of hUCMSC induction on implant osseointegration in an osteoporotic animal model.

**Materials and methods:**

Twenty-eight osteoporotic female Wistar rats were divided into control and hUCMSC-induced groups and observed at 2 and 4 weeks. The treatment group received hUCMSC injections into the implant area of the femur osteoporotic model. Specimens were stained using immunohistochemical and haematoxylin and eosin techniques to assess osteogenic marker expression. Data were analysed using Kruskal–Wallis and Mann–Whitney U test.

**Results:**

Bone-implant volume (BIV) was significantly greater in the hUCMSC-induced groups compared to controls. The expression of osterix, RUNX family transcription factor 2 (RUNX2), tumor necrosis factor (TNF)-α, nuclear factor of activated T cells 1 (NFATc1), collagen type 1, and osteocalcin decreased in osteoblasts from week 2 to week 4. Significant differences (*P* < 0.05) were observed between control and hUCMSC groups at both time points. These findings suggest that bone formation was completed by week 4, entering the bone maturation phase, supported by the increased BIV in the hUCMSC group.

**Conclusion:**

Induction with hUCMSCs promotes both early and late osseointegration in osteoporotic animal models. These results highlight the efficacy of hUCMSCs in enhancing bone healing after implant placement under osteoporotic conditions.

## Introduction

As women age, changes in oestrogen and progesterone production cause the cessation of reproductive capability.^1^ Menopause is defined as the permanent cessation of menstruation for 12 consecutive months owing to inactive ovarian follicles.^2^ Deficiencies in oestrogen and progesterone may affect women's physical, psychological, and emotional health.^3^ Additionally, lower oestrogen levels increase bone mass sensitivity to parathyroid hormone, decrease calcitonin production, and inhibit calcium and vitamin D absorption. These changes result in decreased bone mass, leading to osteoporosis.^4^

Osteoporosis is characterised by damage to bone tissue and microarchitecture, causing bones to become more prone to fracture.^5^ Research from Europe and the United States indicates that 30 % of women worldwide suffer from osteoporosis, with 40 % of these being postmenopausal women.^6^ In Indonesia, women have a 21.7 % increased risk of developing osteoporosis than men, who have a risk of 14.8 %. In 2019, the estimated prevalence of osteoporosis among older women ranged from 22 % to 55 %.^7^ Jawbone osteoporosis may exacerbate alveolar bone resorption, increasing the risk of dental implant failure. This finding is particularly significant because dental implants are the most commonly used restorative option for patients with partial or complete tooth loss.^8^^,^^9^

The success of dental implant treatment depends on a strong structural and functional association between the implant surface and surrounding tissue through a process called osseointegration. Osseointegration is necessary to achieve mechanical support, ensuring that the dental implant is properly anchored in the alveolar bone.^10^ Osteogenic markers associated with bone regeneration include osterix (Osx), RUNX2, collagen type 1, and osteocalcin, all of which are essential for osteoblast differentiation and bone matrix formation. NFATc1 is a key regulator of bone remodelling, balancing osteoblast-mediated bone formation and osteoclast-mediated bone resorption.^11^ TNFα plays a concentration- and cell type-dependent role in bone remodelling: at low levels, it activates osteogenic signalling pathways and stimulates osteoblast precursor proliferation; at higher concentrations, it inhibits osteoblast differentiation and promotes osteoclastogenesis.^12^, ^13^, ^14^ Furthermore, long-term implant stability depends on the underlying bone structure supporting the implant, which is established through bone-implant volume (BIV) formation.

The disparity between the osteoporotic condition and the success of implant placement stems from the propensity of mesenchymal stem cells to preferentially differentiate into adipocytes rather than osteoblasts. A cell-based therapeutic strategy or the administration of exogenous stem cells is therefore expected to induce host cells to undergo differentiation in accordance with their physiological roles. For cell-based therapy, mesenchymal stem cells are used widely due to their multipotent capacity, immunomodulatory effects, and regenerative versatility.^15^ Bone marrow-derived MSCs (BMMSCs) are currently used to promote bone formation in dental implant treatments for osteoporosis. However, BMMSC use is controversial owing to the invasive, strenuous specimen collection procedures, which carry a high morbidity rate.^16^ Therefore, alternative therapies using human umbilical cord MSCs (hUCMSCs) have been developed.^9^

hUCMSCs are osteoprogenitor stem cells capable of promoting bone formation.^17^ They offer several advantages over other MSC types, including simple specimen isolation, no requirement for invasive procedures, absence of ethical concerns, an abundant umbilical cord supply, and the ability to be cultured in large quantities in vitro.^18^ Administration of hUCMSCs in osteoporotic rat models yielded satisfactory results. These include increased osteoblast number and differentiation, as well as elevated expression of bone formation markers such as transforming growth factor-β1, Runt-related transcription factor 2 (Runx2), alkaline phosphatase (ALP), collagen type I, osteocalcin, Osx, and bone morphogenetic protein 2 (BMP-2).^19^^,^^20^

Studying hUCMSCs as an alternative therapy for bone regeneration under osteoporotic conditions is important for elucidating the mechanisms by which hUCMSCs promote osteogenesis, identifying key molecular pathways involved in bone regeneration, and demonstrating their potential to accelerate healing. Moreover, hUCMSCs offer a minimally invasive therapeutic approach with low immunogenicity, no risk of graft rejection, and no donor site morbidity, supporting their effective clinical application in regenerative medicine.

Numerous studies have investigated strategies to enhance osseointegration in osteoporotic bone, with a primary focus on mechanical interventions. Most of the inventions involve implant surface modifications such as **micro and nano roughening or** bioactive coatings. While these surface modifications may enhance initial implant stability, they tend to promote bacterial adhesion and biofilm formation, thereby increasing susceptibility to peri-implant disease.^21^

This shortage underscores the need of a cell-based therapeutic strategy employing hUCMSCs, which show considerable promise in enhancing peri-implant osseointegration and increasing peri-implant bone density, thereby improving long-term prognostic outcomes. Therefore, this study was conducted to examine BIV, TNFα, and osteogenic markers at 2 and 4 weeks after implant placement with hUCMSC induction in experimental osteoporotic animal models to evaluate implant osseointegration.

## Materials and methods

This research was approved by the General Hospital of Dr. Soetomo Surabaya, Indonesia (approval for donor informed consent: 547/Panke.KKE/IX/2017), and by the Ethical Commission of the Faculty of Veterinary Medicine (2.KE.152.09.2018). The animal laboratory used for this study included 28 three-month-old female Wistar strain *Rattus norvegicus* Albinus rats weighing 180–200 g. The number of animals was determined using the Lemeshow formula based on data from a previous study.^22^ The animals were divided into four groups: ovariectomy groups injected with gelatin solvent for 2 weeks (C1) or 4 weeks (C2), and ovariectomy groups injected with hUCMSCs and gelatin for 2 weeks (P1) or 4 weeks (P2).

### hUCMSCs isolation, culture, and characterisation

The umbilical cord was obtained from the placenta of a healthy, full-term baby delivered by elective Caesarean section without medical complications. The umbilical cord was cut into approximately 1 cm segments, and the artery, vein, and adventitia were separated to isolate Wharton's Jelly. Wharton's Jelly was then sliced with a scalpel into approximately 1 mm^3^ sections and used as the primary culture source of hUCMSCs.

Characterisation of the hUCMSCs phenotype was performed using flow cytometry, hUCMSCs were seeded in wells with Alpha Minimum Essential Medium, then fixed with 10 % formaldehyde and incubated using the Human MSC Analysis Kit (BD Stemflow™, BD Biosciences, Piscataway, NJ, USA) with primary antibodies mouse anti-human CD73, CD90, CD105.

### Osteoporosis animal model

Female Wistar rats weighing 180–200 g were housed individually for 1 week prior to ovariectomy. Ovariectomy was performed via a ventral incision from the umbilicus to the pubis. The ovaries and fallopian tubes were ligated separately, and bilateral ovaries and periovarian fat were completely removed. The peritoneal incision was closed with simple sutures before closing the skin. Postoperatively, rats were allowed free movement in their cages and fed a normal diet for 12 weeks. The ovariectomy procedure was conducted and monitored by an experienced veterinarian, and rats were observed until full recovery.

### Implant placement on the femur of the animal model

Subjects were fasted for 6–8 h before surgery. Anaesthesia was administered via intramuscular injection of 10 % ketamine (1 cc) and xylazine (1 cc) into the semitendinosus muscle. The fur on the femur where the implant was placed was shaved and cleaned with iodine compound and 80 % alcohol. The instruments used were sterilized in an autoclave. A 10 mm incision was made from the femur's dorsal surface to the bone. Drilling was performed 7 mm from the distal edge of the femur, corresponding to implant length and diameter, with saline irrigation at 800 rpm and a torque of 20 N.^23^ The implant was placed on the mesial surface osteotomy of the femur, and primary stability was ensured before suturing muscles and skin with 4-0 Vicryl. Sutures were removed 7 days post-implantation.

### hUCMSCs injection into the implant area of femur osteoporotic animal model

A perforation was made in the femur at the implant site using a needle perforator (Stabident Intraosseous System, Fairfax Dental Inc, Miami, FL, USA) to penetrate the bone. After removal of the needle, hUCMSCs suspended in gelatin solvent (P1 and P2 groups) or gelatin solvent alone (C1 and C2 groups) were injected into the perforation site using a 1 mL syringe.

### Termination of experimental animals and specimen preparation

Anaesthesia was administered via intramuscular injection of 10 % ketamine and xylazine (1 cc), followed by perfusion. An incision was made around the implant site on the femur, and a 0.5 mm margin mesial and distal to the implant was cut. The specimen was rinsed with phosphate-buffered saline (PBS) and fixed in 10 % formalin. It was then washed sequentially with graded alcohol concentrations and cleared with xylene. The tissue was infiltrated with soft paraffin, embedded, and sectioned into 4–6 μm-thick slices. Sections were mounted onto glass slides for further analysis.

### Immunohistochemical staining

Endogenous peroxidase activity was quenched by incubation with 3 % hydrogen peroxide for 10 min, followed by washing with PBS. The sections were incubated with 0.025 % trypsin at 37 °C for 6 min, then washed with double-distilled water. Ultra V Block was added and incubated for 5 min. Primary antibodies—RUNX2 (Santa Cruz Biotechnology, Santa Cruz, CA, USA), COL1 (Santa Cruz Biotechnology), NFATc1 (Santa Cruz Biotechnology), TNF-α (Santa Cruz Biotechnology), Osx (Abcam, Cambridge, UK), and osteocalcin (Santa Cruz Biotechnology)—were applied at optimised dilutions and incubated. After washing with PBS, these antibodies were used due to their roles in osteogenesis, inflammation, and angiogenesis, which are critical to bone formation and rem odelling.

Primary Antibody Enhancer (Thermo Scientific, Fremont, CA, USA) was applied next, incubated for 15 min at room temperature, and washed with PBS. Secondary antibodies conjugated to horseradish peroxidase were then incubated for 15 min at 18 °C. The reaction was visualised using diaminobenzidine substrate, producing a brown precipitate marking antigen–antibody complex. Haematoxylin was used as a counterstain to visualise nuclei. Finally, sections were dehydrated, cleared, and mounted with coverslips.

Expression levels of target proteins were evaluated under a light microscope. Staining intensity was semi-quantitatively scored based on the Remmele scale index (immunoreactive score), calculated by multiplying the immunoreactive cell percentage score by the staining intensity score in immunoreactive cells (Table 1).

**Table 1:** IRS (Immunoreactive score) as established by Remmele and Stegner and modified by Halon et al.^39^

Percentage of positive cells	Points	IRS (Immunoreactive Score) Intensity of reaction	Points
No positive cells	0	No reaction	0
<25 % positive cells	1	Weak colour reaction	1
25–50 % positive cells	2	Moderate intensity	2
51–75 % positive cells	3	Intense reaction	3
>75 % positive cells	4		

### Statistical analysis

Data were analysed to compare groups. For non-normally distributed data, the Kruskal–Wallis test was used as the primary non-parametric method. Subsequent pairwise comparisons were performed using the Mann–Whitney U test. A *P*-value <0.05 was considered statistically significant. All analyses were conducted using IBM SPSS Statistics 25 (IBM Corp., Armonk, NY, USA).

## Results

### Flowcitometry of hUCMSCs

**Figure 1:**
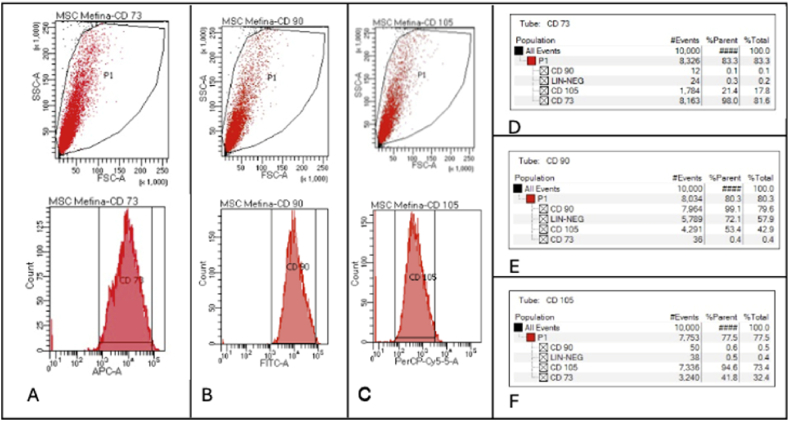
Fluorescence-activated cell sorting (FACS) plots, expression percentage and gating strategy of hUCMSCs. (A) Positive cluster of differentiation (CD) 73; (B) Positive CD 90; (C) Positive 105; (D) Expression percentage and gating strategy of CD 73; (E) Expression percentage and gating strategy of CD 90; (F) Expression percentage and gating strategy of CD 105.

Characterisation of the hUCMSCs phenotype demonstrated positivity for CD73, CD90, and CD105 markers (Fig. 1).

### BIV in rat femur bone

**Figure 2:**
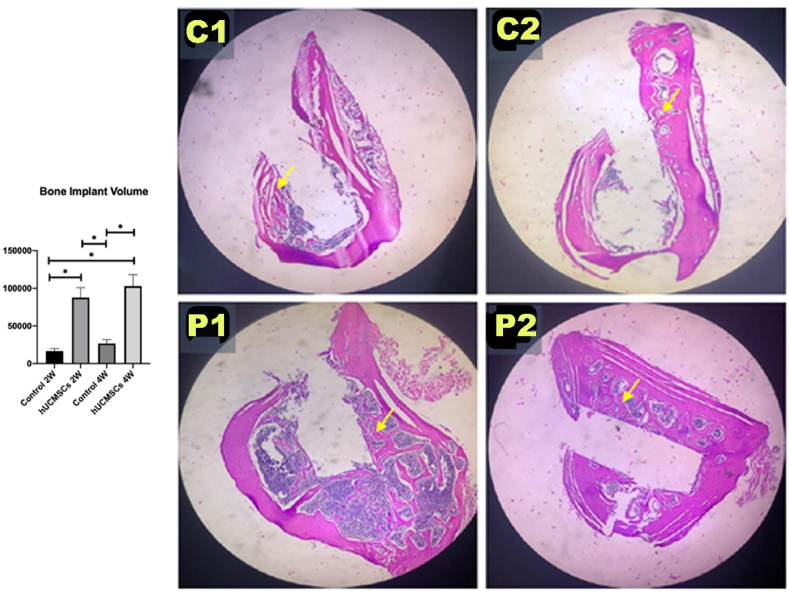
Graph analysis of bone-implant volume (BIV) and histological image of each experimental group for BIV. Single asterisk (∗) indicates a statistically significant difference (*P*-value <0.05), n = 7. Yellow arrow indicates a new bone formation around the implant surface with 40 × magnification. C1: control 2 weeks; C2: control 4 weeks; P1: human umbilical cord mesenchymal stem cells (hUCMSCs) induction 2 weeks; P2: hUCMSCs induction 4 weeks.

Fig. 2 shows BIV results measured based on the volume of new bone formation around the cortical surface of the implant. The highest BIV was found in group P2, followed by P1 and C2. The lowest BIV was found in group C1.

### Expression of Osx and Runx2 in rat femur bone

**Figure 3:**
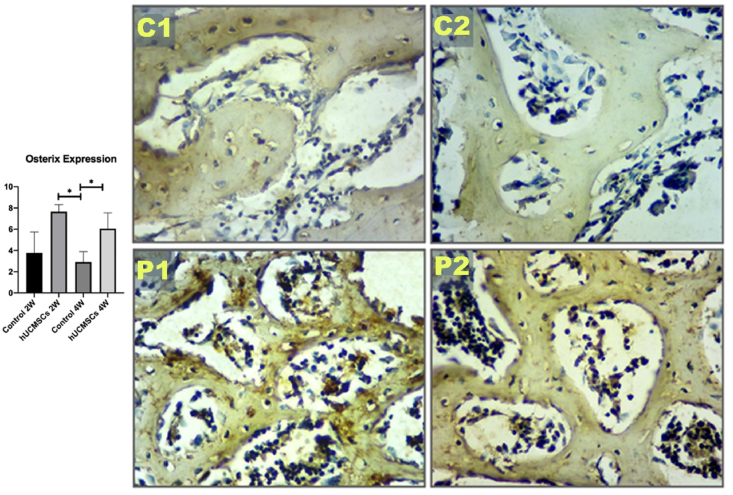
Graph analysis of osterix (Osx) expression and Osx immunohistochemical image of each experimental group with 400 × magnification. Single asterisk (∗) indicates a statistically significant difference (*P*-value <0.05), n = 7. C1: control 2 weeks; C2: control 4 weeks; P1: human umbilical cord mesenchymal stem cells (hUCMSCs) induction 2 weeks; P2: hUCMSCs induction 4 weeks.

**Figure 4:**
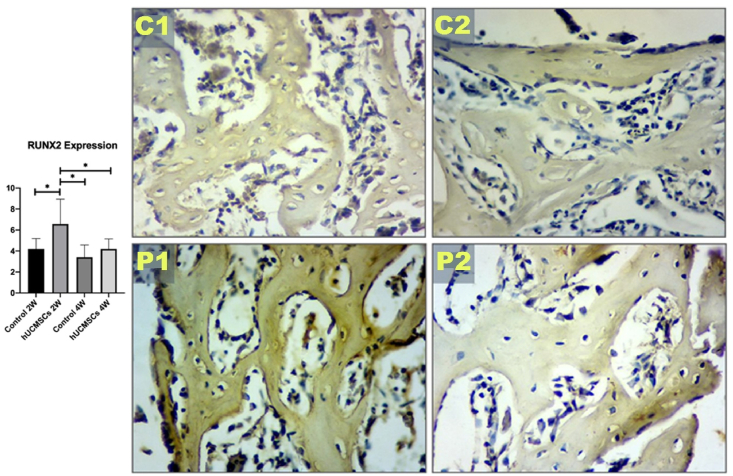
Graph analysis of Runt-related transcription factor 2 (Runx2) expression and Runx2 immunohistochemical image of each experimental group with 400 × magnification. Single asterisk (∗) indicates a statistically significant difference (*P*-value <0.05), n = 7. C1: control 2 weeks; C2: control 4 weeks; P1: human umbilical cord mesenchymal stem cells (hUCMSCs) induction 2 weeks; P2: hUCMSCs induction 4 weeks.

Osx and Runx2 expressions were evaluated using immunohistochemical staining with chromogenic colour in osteoblasts. The highest expression of Osx and Runx2 was observed in group P1, followed by P2 and C1. The lowest expression was in group C2 (Figure 3, Figure 4).

### Expression of TNF-α in rat femur bone

**Figure 5:**
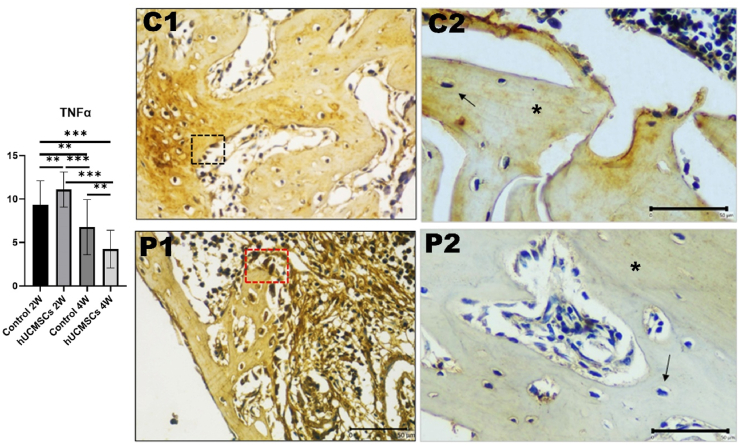
Graph analysis of tumor necrosis factor α (TNFα) and TNFα immunohistochemical image of each experimental group with 400 × magnification. Each group had significant differences (*P*-value <0.05). Triple asterisks (∗∗∗) indicates a statistically significant difference (*P*-value <0.001). Comparison of TNFα expression in osteoblast (inlet), osteocyte (arrow) and bone matrix (asterisk) between treatment groups. The results of this study showed that TNFα expression of P2 group is the lowest compared to other groups (Immunohistochemical staining, 40 × objective lens; bar = 50 μm; microscope Nikon Eclipse E-i; camera DS Fi2 300 megapixel; C1: control 2 weeks; C2: control 4 weeks; P1: human umbilical cord mesenchymal stem cells (hUCMSCs) induction 2 weeks; P2: hUCMSCs induction 4 weeks.

TNF-α expression decreased from week 2 to week 4, similar to all other markers except BIV. Significant differences were observed between C1 and P1, C2, and P2 groups (*P* < 0.01, *P* < 0.01, and *P* < 0.001, respectively). The treatment groups showed the highest expression at week 2 and the lowest at week 4, with *P* < 0.001 (Fig. 5).

### Expression of NFATc1 in rat femur bone

**Figure 6:**
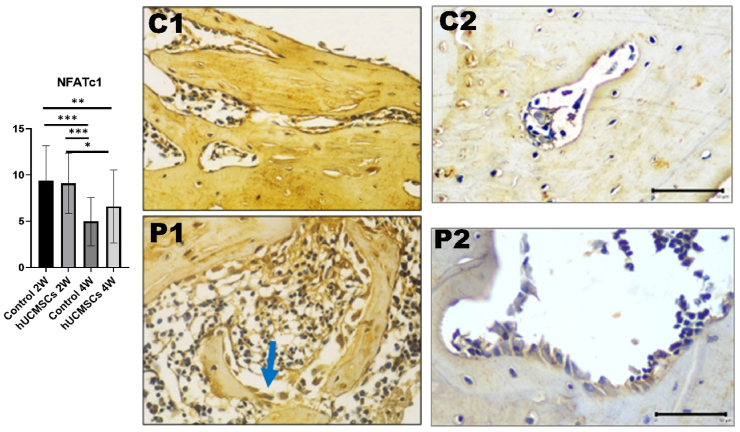
Graph analysis of nuclear factor of activated T-cells, cytoplasmic 1 (NFATc1) and comparison of NFATc1 immunohistochemical image expression between treatment groups. Each group had significant differences (*P*-value <0.05). Triple asterisks (∗∗∗) indicates a statistically significant difference (*P*-value <0.001). In bone, NFATc1 is secreted by osteoblasts and other osteogenic cells and mononuclear white blood cells. The above slide shows osteoblast cells with NFATc1 expression (chromogen brown colour) (Immunohistochemical staining, 40 × objective lens; bar = 50 μm; Microscope Nikon Eclipse E-i; Camera DS Fi2 300 megapixel; C1: control 2 weeks; C2: control 4 weeks; P1: human umbilical cord mesenchymal stem cells (hUCMSCs)induction 2 weeks; P2: hUCMSCs induction 4 weeks.

No significant differences were found between C1 and P1 or between C2 and P2. Significant differences were observed between C1 and C2 (*P* < 0.001) and between C1 and P2 (*P* < 0.01). The reduction in expression from 2 to 4 weeks was significantly more pronounced in the control group than in the treatment group (*P* < 0.001 and *P* < 0.05, respectively) (Fig. 6).

### Expression of collagen type 1 in rat femur bone

**Figure 7:**
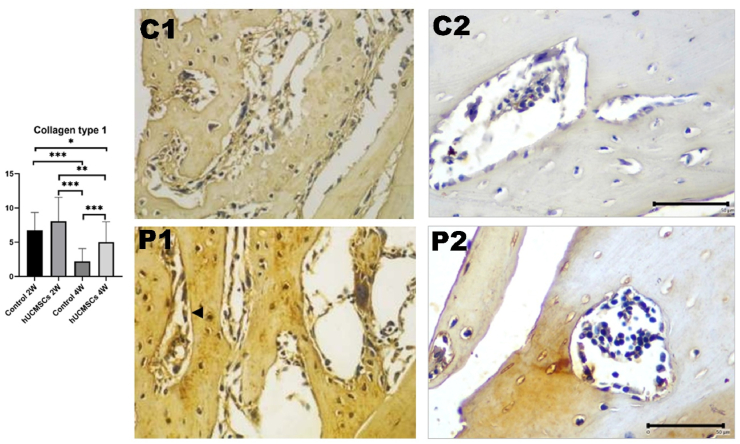
Graph analysis of collagen type 1. Each group had significant differences (*P* < 0.05). Triple asterisks (∗∗∗) indicates a statistically significant difference (*P*-value <0.001). Collagen type 1×-expressing osteoblast cells are indicated by arrows and appear in a chromatic brown colour (immunohistochemical staining, 400 × magnification; inlet 1000 × microscope Nikon H600L; DS Fi2 300-megapixel camera, C1: control 2 weeks; C2: control 4 weeks; P1: hUCMSCs induction 2 weeks; P2: hUCMSCs induction 4 weeks.

The highest collagen type 1 expression was in P1, followed by C1, P2, and C2 (Fig. 7). A significant difference was found between C2 and P2 (*P* < 0.001), but not between C1 and P1. A downward trend in collagen type 1 expression was observed from week 2 to week 4, both between C1 and C2 (*P* < 0.001) and between P1 and P2 (*P* < 0.01).

### Expression of osteocalcin in rat femur bone

**Figure 8:**
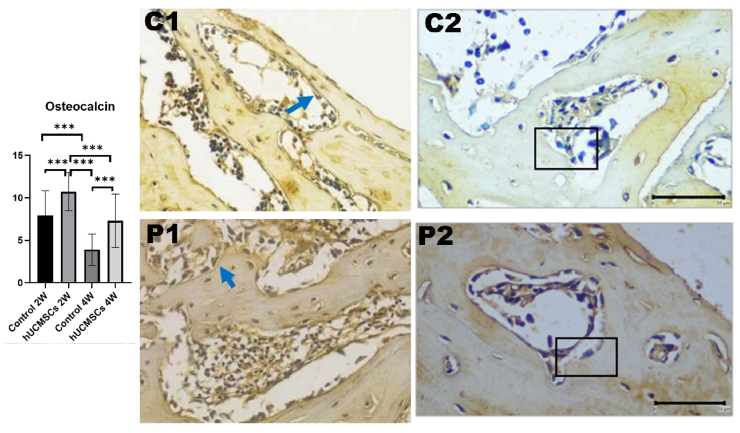
Graph analysis of osteocalcin and comparison of osteocalcin immunohistochemical image expression between treatment groups. Each group had significant differences (*P*-value <0.05). Triple asterisks (∗∗∗) indicates a statistically significant difference (*P*-value <0.001). In bone, osteocalcin is secreted only by osteoblast cells (inlet). The above slide shows osteoblast cell with osteocalcin expression (chromogen brown color) (Immunohistochemical staining, 40 × objective lens; inlet 100 objective lens; bar = 50 μm; Microscope Nikon Eclipse E-i; Camera DS Fi2 300 megapixel; C1: control 2 weeks; C2: control 4 weeks; P1: hUCMSCs induction 2 weeks; P2: hUCMSCs induction 4 weeks.

Osteocalcin expression increased in treatment groups compared to controls at weeks 2 and 4. Significant differences were observed between C1 and P1 (*P* < 0.001) and between C2 and P2. The increase was more pronounced at week 2 than week 4. A significant difference was also seen between P1 and P2, with week 4 exhibiting lower expression than week 2 (*P* < 0.001) (Fig. 8).

## Discussion

Osteoporosis, characterized by decreased bone mineral density and microarchitectural deterioration, poses significant challenges to dental implantology. Although some studies suggest that osteoporosis may not directly influence implant failure rates, the altered bone quality associated with this condition can impact the initial stability and long-term success of dental implants. Studying the mechanisms of cell-based therapy administration in implants under osteoporotic conditions is crucial for achieving favorable clinical outcomes.

In osteoporotic conditions, precise modulation of TNF-α is crucial to leverage its beneficial effects on bone healing while mitigating potential adverse outcomes. Excessive TNF-α activity, where bone turnover is already elevated, can exacerbate bone loss and delay healing. In the early stages of bone healing, TNF-α plays a crucial role in initiating the inflammatory response, clearing necrotic tissue, and preparing the site for new bone formation. As healing progresses into the reparative phase, the inflammatory response should subside, leading to a decrease in TNF-α levels.^24^^,^^25^ In this study, a significant increase in TNF-α levels was observed in the treatment group at 2 weeks, whereas at 4 weeks, a significant decrease was noted compared to the control group. This indicates the potential of stem cell therapy in modulating TNF-α activity. In osteoporotic conditions, careful regulation of TNF-α is essential to balance the pro-inflammatory and anti-inflammatory phases, thereby promoting successful osseointegration.

RUNX2 is a pivotal transcription factor that initiates the differentiation of mesenchymal stem cells into preosteoblasts, while Osterix (Osx), functioning downstream of RUNX2, is essential for the maturation of osteoblasts and the subsequent mineralization of the extracellular matrix. In osteoporotic conditions, the expression levels of both RUNX2 and Osx are diminished, which impairs the osseointegration process. This reduction in expression hinders the formation of mature osteoblasts and the deposition of mineralized bone matrix, thereby compromising the osseointegration of dental implants into the surrounding bone tissue.^26^^,^^27^ The administration of hUCMSCs in this study yielded statistically significant outcomes, as evidenced by the highest expression levels of RUNX2 and Osx in group P1, which differed significantly from those observed in the other groups. This indicates that stem cell administration exerts a substantial effect in elevating RUNX2 and Osx levels.

Transcription factors involved in late osteogenesis regulate osteoblast differentiation and mineralisation, including osteopontin, osteocalcin, bone sialoprotein (BSP), and others. Osteocalcin and BSP are non-collagenous bone proteins, with osteocalcin being most abundant. Increased osteocalcin levels correlate with higher bone mineral density^28^ Osteocalcin, a late-stage osteogenic marker, showed decreased expression by week 4, suggesting that the peak of osteoblast differentiation occurred before week 2, resulting in a downward trend in all osteogenic markers from week 2 to week 4. Overexpression of Osx increases ALP and osteocalcin expression, leading to bone tissue calcification.^29^^,^^30^

Osteoblasts expressing NFATc1 modulate the expression of receptor activator of nuclear factor kappa-B ligand and osteoprotegerin, two key molecules involved in regulating osteoclastogenesis. Thus, NFATc1 serves as a mediator of homeostasis between bone formation and resorption.^11^ During osteoblast differentiation, NFATc1 cooperates with Osx as a crucial transcriptional partner.^31^ Mice expressing nuclear-localised NFATc1 in osteoblasts exhibited a marked increase in bone mass after 4 weeks, accompanied by enhanced osteoblast proliferation. The mechanism by which NFATc1 regulates osteoblast proliferation involves a modest enhancement of its nuclear occupancy.^11^ These findings support the results of the present study, where NFATc1 expression was relatively high at week 2 alongside other osteogenic markers such as RUNX2, collagen type 1, Osx, and osteocalcin. At sufficient concentrations, NFATc1 is hypothesised to facilitate accelerated osteoblast differentiation. NFAT signalling is believed to regulate osteoblast proliferation and function at various stages of osteogenesis.

Higher BIV values correlate with enhanced implant stability, faster healing, and successful osseointegration, as they reflect a greater proportion of mineralized bone contacting the implant surface which is an essential factor for long-term implant success.^32^ In this study, the treatment group exhibited a significant increase in BIV compared to the control group. Notably, even at four weeks, the control group remained significantly different from the two-week treatment group. Consequently, through the evaluation of various biomarkers and BIV as definitive indicators of osseointegration, it can be concluded that cell-based therapy significantly accelerates and enhances the osseointegration process. These findings underscore that MSC strategies not only increase the mineralized bone at the implant interface, a critical determinant of osseointegration quality, but also facilitate faster and more stable implant osseointegration.

Administration of hUCMSCs serves as a source of MSCs to compensate for MSC deficiencies in osteoporosis. An adequate MSC source increases the potential for cell differentiation into osteoprogenitors for bone formation. The initiation of osteoblastogenesis induces formation of a new bone matrix, initially as osteoid (uncalcified bone), which is subsequently mineralised until bone remodelling is complete.^33^ The use of hUCMSCs has demonstrated higher osteogenic differentiation potential, particularly in the expression of ALP and osteocalcin, compared to other stem cell types such as chorionic membrane and decidua MSCs.^34^ In response to bone injury, stem cells induce paracrine signalling through secretion of Wnt-related molecules, which activate BMP and Wnt/β-catenin pathways, leading to osteogenic differentiation.^35^ Furthermore, MSCs create a microenvironment conducive to new bone formation via extracellular matrix production.^34^ MSC administration to the distal femur of osteoporotic rabbits enhances bone formation, trabecular thickness, and overall bone tissue volume.^36^

In treatment groups P1 and P2, gelatin solvent and hUCMSCs were locally injected at the implant site. Huang et al. (2015) reported that localMSC injection supports healing and bone remodelling similarly to systemicMSC administration, without eliciting immune rejection in experimental animals. The study also indicated that localMSC application is preferred for supporting bone formation (osteogenesis) in simple cases not involving multiple fractures.^37^ Locally injected MSCs directly contribute to bone repair at the implant site. This result is supported by other studies showing that MSCs have multipotent differentiation capacity and can survive conditions such as hypoxia to aid tissue repair.^38^

The findings suggest that cell-based therapy for osteoporosis yields promising outcomes with limited adverse effects, attributable to its localized delivery. This approach distinguishes itself from other treatments, such as biomaterial implant development, which is associated with higher costs, limited accessibility, and a potential increase in side effects like peri-implantitis. Additionally, systemic approaches, including bisphosphonate or hormone therapy, can have negative impacts on overall health, further highlighting the potential benefits of localized cell-based therapies.

In conclusion, despite the limitations of this study, it can be concluded that localized stem cell administration can accelerate and enhance osseointegration in osteoporosis conditions through various evaluated markers Osx, osteocalcin, collagen type 1, RUNX2, NFATc1, TNFα, and BIV.

## Declaration of competing interest

The authors declare no conflicts of interest relevant to this article.
