# Supplementary material for: Immunohistochemical evaluation of bone regeneration induced by human umbilical cord mesenchymal stem cells around implants in an osteoporotic rat model
Source: J Dent Sci. 2025 Sep 9;21(1):294–304. doi: 10.1016/j.jds.2025.08.042 (PMC12825505; doi:10.1016/j.jds.2025.08.042)
Supplement: Multimedia component 1 [file mmc1.pdf]

Certificate of Elsevier Language Editing Services

The following article was edited by Elsevier Language Editing Services:

Immunohistochemical evaluation of human umbilical cord mesenchymal stem cells-induced bone regeneration around implant in an osteoporotic rat model

Ordered by:

mefina kuntjoro

Estimated Delivery date:

2025-07-11

Order reference:

ASLESTD1108423
